# Genomic organization and phylogenetic utility of deer mouse (*Peromyscus maniculatus*) lymphotoxin-alpha and lymphotoxin-beta

**DOI:** 10.1186/1471-2172-9-62

**Published:** 2008-10-31

**Authors:** Tiffany Richens, Aparna D~N Palmer, Joseph Prescott, Tony Schountz

**Affiliations:** 1 Department of Biological Sciences, Mesa State College, Grand Junction, Colorado, 81502, USA; 2 Center for Infectious Diseases and Immunity, Department of Pathology, University of New Mexico School of Medicine, Albuquerque, NM 87131, USA; 3 School of Biological Sciences, University of Northern Colorado, Greeley, CO 80639, USA

## Abstract

**Background:**

Deer mice (*Peromyscus maniculatus*) are among the most common mammals in North America and are important reservoirs of several human pathogens, including Sin Nombre hantavirus (SNV). SNV can establish a life-long apathogenic infection in deer mice, which can shed virus in excrement for transmission to humans. Patients that die from hantavirus cardiopulmonary syndrome (HCPS) have been found to express several proinflammatory cytokines, including lymphotoxin (LT), in the lungs. It is thought that these cytokines contribute to the pathogenesis of HCPS. LT is not expressed by virus-specific CD4^+ ^T cells from infected deer mice, suggesting a limited role for this pathway in reservoir responses to hantaviruses.

**Results:**

We have cloned the genes encoding deer mouse LTα and LTβ and have found them to be highly similar to orthologous rodent sequences but with some differences in promoters elements. The phylogenetic analyses performed on the LTα, LTβ, and combined data sets yielded a strongly-supported sister-group relationship between the two murines (the house mouse and the rat). The deer mouse, a sigmodontine, appeared as the sister group to the murine clade in all of the analyses. High bootstrap values characterized the grouping of murids.

**Conclusion:**

No conspicuous differences compared to other species are present in the predicted amino acid sequences of LTα or LTβ; however, some promoter differences were noted in LTβ. Although more extensive taxonomic sampling is required to confirm the results of our analyses, the preliminary findings indicate that both genes (analyzed both separately and in combination) hold potential for resolving relationships among rodents and other mammals at the subfamily level.

## Background

Lymphotoxins-α (LTα) and -β (LTβ) are members of the tumor necrosis factor (TNF) superfamily and are closely linked to the TNF gene within the major histocompatibility complex of all known mammalian species. LTα polypeptides can form a soluble homotrimer (LT) that is secreted principally by activated T lymphocytes and NK cells, while LTβ polypeptides form type II membrane-bound heterotrimers with LTα polypeptides (LTα_1_β_2 _or LTα_2_β_1_) [1]. Each form of the trimer is involved in inflammatory responses, induction of apoptosis of target cells, and lymphoid organogenesis.

Hantavirus cardiopulmonary syndrome (HCPS) is caused by several pathogenic New World hantaviruses, but most North American cases of HCPS are caused by Sin Nombre virus (SNV) [2-5]. The disease is characterized by the production of several proinflammatory cytokines, including LT, in the lungs [6]. In contrast, the principal reservoir host of SNV, the deer mouse (*Peromyscus maniculatus*) [7], exhibits no conspicuous pathology and remains persistently infected for life [8,9]. It is unknown how deer mice can be persistently infected without pathology because few methods have been developed for characterizing cytokine responses in this species. We previously reported a partial cDNA sequence of deer mouse LTα [10] and present here genomic, structural, and phylogenetic characterizations of the complete lymphotoxin genes.

As one of the most ecologically and morphologically diverse groups within Mammalia, Rodentia has always been a challenge to delineate systematically. And, despite the attention the group has received from ecologists and evolutionary biologists, the lineages within the group have been difficult to resolve. In particular, the relationships among members of the family Muridae, arguably the most species-rich family of mammals, have yet to be definitively resolved at the subfamily level and below [11]. The introduction and analysis of new sources of molecular data, such as those sampled here, may represent an avenue for understanding the evolution and diversification of the rodents. In addition, the prospect of combining data sets, as examined here, allows investigators a greater suite of options when it comes to understanding the relationships within highly diverse groups such as the Muridae family. Although the taxonomic sampling within this study was limited by the availability of orthologs in GenBank, we have conducted a preliminary examination of the usefulness of LTα and LTβ for phylogeny reconstruction at the subfamily level within the order Rodentia. We present here the extent to which these lymphotoxins (both in separate and combined data sets) have potential for resolving relationships within Muridae.

## Results

### Deer mouse *Lta *gene and polypeptide

**Figure 1:**
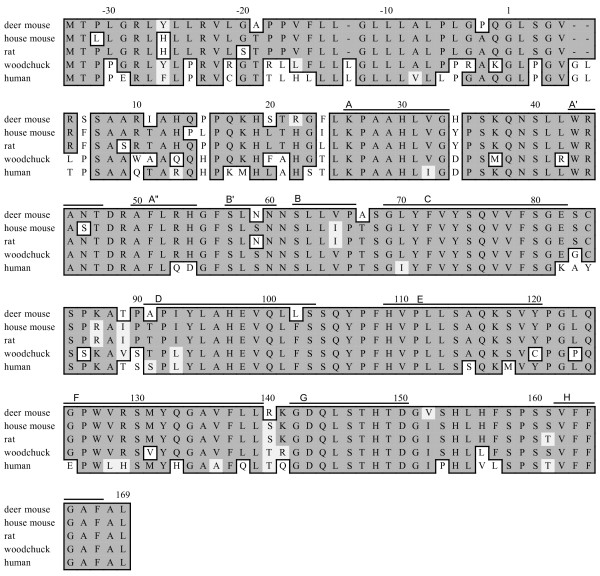
**Amino acid alignment of rodent and human LTα polypeptides.** Sequences were aligned with MacVector software. Identical (dark shading) and similar (light shading) residues are framed. β-strands that form β-sheets are noted by bars above the alignment.

The *Lta *sequence is 1,085 nucleotides (nt) and includes partial 5' and 3' untranslated regions (UTR). No *cis*-elements were identified in the UTRs due to insufficient sequence information. As with other *Lta *genes, deer mouse *Lta *is composed of three exons. The putative LTα polypeptide is highly conserved with LTα from all other known rodent species (Fig. 1). It possesses a 33 residue leader sequence, and is 92% similar and 90% identical to house mouse (*Mus musculus*) LTα [12]. The β-strands that form the inner (A", A, C, F, H) and outer (A', B', B, D, E, G) β-sheets common to TNF family members are present [13]. D48 in the A'-A" loop and Y106 in the D-E loop involved in binding to TNFR1 [14] are conserved in deer mouse LTα.

### Deer mouse *Ltb *gene and polypeptide

**Figure 2:**
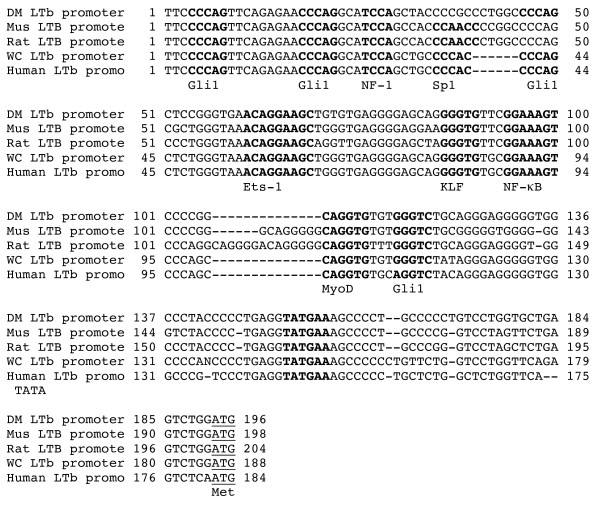
***Cis*****-elements in the promoter of the deer mouse *****Ltb *****gene.** Boldface underlined sequences represent putative *cis*-elements with the names of the transcription factor. The underlined ATG indicates the start codon for each ortholog.

The assembled *Ltb *gene is 1,840 nt and promoter analysis (NIH Signal Scan) revealed several putative 5' UTR *cis*-elements, including NF-1, Krüppel-like factor (KLF), Ets-1, NF-κB and MyoD and several Gli1 sites (Fig. 2) similar to that of orthologous *Ltb *[15,16]. The deer mouse gene uniquely possesses an Sp1 site. An unconventional TATA signal identical to that in house mouse *Ltb *[16] is relatively close to the ATG start codon, suggesting the mRNA has a short 5' UTR. The CCCAG motif, a putative site for Gli transcription factors that are thought to control adult organ sizes [17], occurs at four identical sites in the deer mouse and house mouse, four sites in the human, and five sites in the rat (*Rattus norvegicus*) and woodchuck (*Marmota monax*) sequences.

**Figure 3:**
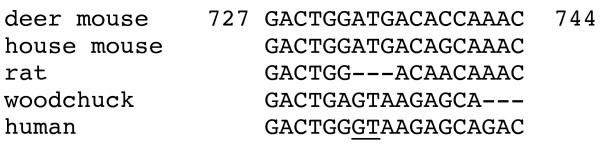
**Exon 2 splice variation between rodent and human Ltb genes.** Human *LTB *possesses at the end of exon 2 a splice donor site (underlined) that is missing in rodent orthologs. Deer mouse and house mouse genes have an A at this position, while the rat has a 3 nt deletion. Although woodchuck gene has a GT at this position, it does not appear to have the surrounding consensus sequence to support splicing. For this reason, rodent LTβ are substantially larger than human.

The deer mouse gene also has an A at nt 733 that is found in house mouse *Ltb *(Fig. 3). In human *LTB*, this nucleotide is a G and acts as a donor splice site that results in a four exon gene. In the four rodent species for which *Ltb *sequences are available, the genes contain only three exons because the intron 2 found in the human sequence encodes additional amino acids in the rodent sequences. Both deer mice and house mice have the A at this position, while the rat possesses a 3 nt deletion that encompasses this location. Interestingly, the woodchuck sequence has the same GT donor splice site as the human sequence, but apparently does not have a consensus splice signal and therefore has three exons like the other rodent genes [18].

**Figure 4:**
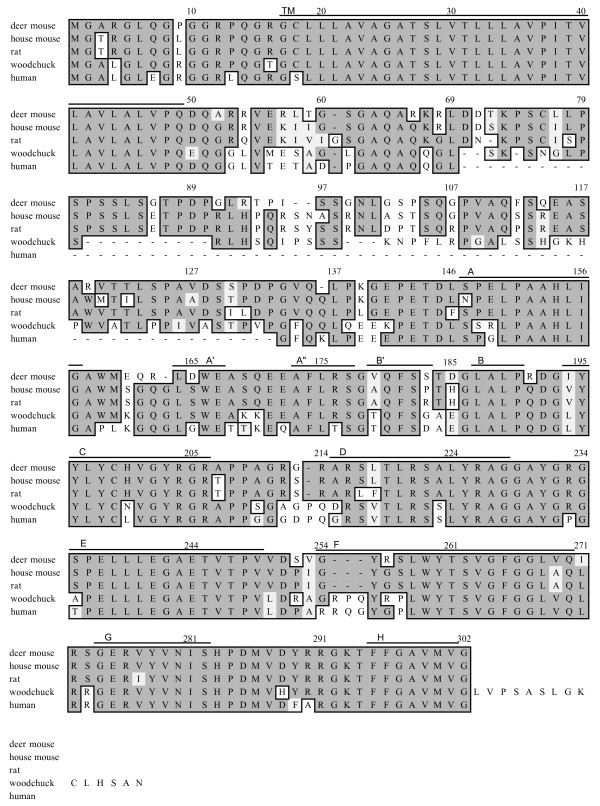
**Amino acid alignment of rodent and human LTβ polypeptides.** Sequences were aligned and denoted as described in Fig. 1.

The putative deer mouse LTβ polypeptide is composed of 302 amino acids and shares 88% similarity and 84% identity with house mouse LTβ (Fig 4). It possesses a 33 residue transmembrane domain that is completely conserved among all four rodent LTβ polypeptides. The β-strands that form the β-sheets of the polypeptide are well-conserved in deer mouse LTβ. Human LTβ is shorter due to the presence of the additional intron. This region is predicted to control the distance of the TNF domains from the plasma membrane, with rodent LTβ protruding further outward, by virtue of the extra amino acids, forming a stalk [16].

### Phylogenetic analysis of LTα sequences

**Figure 5:**
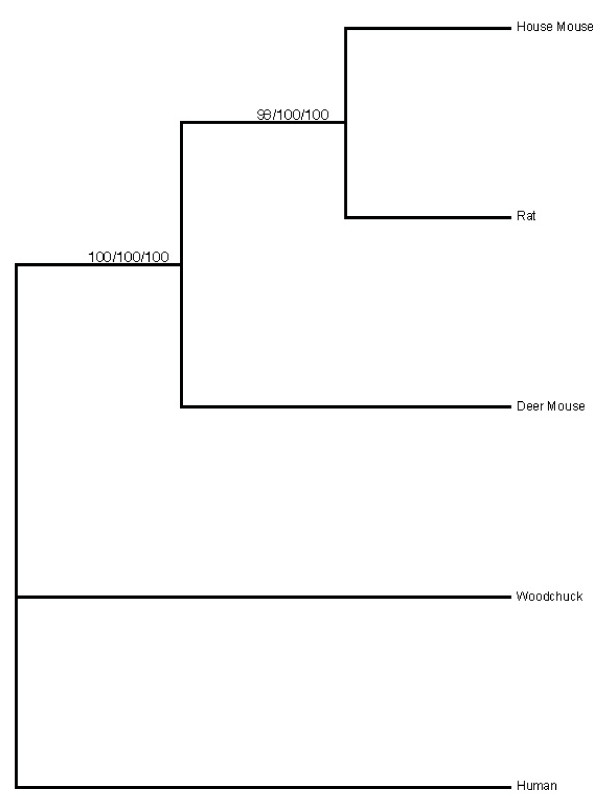
**Maximum parsimony tree resulting from the LTα, LTβ, and combined analyses.** The numbers (left to right) above the branches represent bootstrap values for the LTα, LTβ, and combined analyses, respectively.

The final alignment of the LTα nucleotide sequences included a total of 571 characters, 116 of which were variable but not parsimony-informative, and 66 of which were parsimony-informative. The single most parsimonious tree resulting from the analysis of this data set (C.I. excluding uninformative characters = 0.9067; R.I. = 0.6818; length = 225 steps) showed a sister-group relationship between the house mouse and the rat, both of which are in the subfamily Murinae (Fig. 5). This clade was well-supported by a bootstrap value of 99%. Sister to this clade of murine rodents was the deer mouse (traditionally, a member of the subfamily Sigmodontinae); this well-resolved grouping of murid rodents was supported by a bootstrap value of 100%.

### Phylogenetic analysis of LTβ sequences

The lymphotoxin-beta alignment included a total of 900 characters, 358 of which were variable but not parsimony-informative, and 131 of which were parsimony-informative. The single most parsimonious tree resulting from this analysis (C.I. excluding uninformative characters = 0.8687; R.I. = 0.8015; length = 647 steps) also showed a sister-group relationship between the house mouse and the rat, supported by a bootstrap value of 100% (Fig. 5). Sister to the house mouse-rat clade was the deer mouse lineage; the grouping of murids was supported by a bootstrap value of 100%.

### Phylogenetic analysis of combined data set

The results of the partition-homogeneity test indicated that the LTα and LTβ data sets were not significantly incongruent and could, therefore, be combined into a single data set. The combined data set included a total of 1471 characters; of these characters, 474 were variable but not parsimony-informative while 197 were parsimony-informative. As in the case of the separate analyses, the single most parsimonious tree resulting from the combined analysis (C.I. excluding uninformative characters = 0.8396; R.I. = 0.7614; length = 872) showed a well-supported clade (bootstrap value = 100%) consisting of the house mouse and the rat (Fig. 5). Sister to this clade was the deer mouse lineage; the grouping of murid rodents was supported by a bootstrap value of 100%. Finally, while all three analyses reconstructed the traditional subfamily designations within the family Muridae, none of them was able to resolve which of the two outgroups (Sciuridae or Hominidae) was most closely related to the murids.

## Discussion

Lymphotoxin is expressed in fatal cases of HCPS and may contribute to the inflammatory pathogenesis of the disease [6]. In lymphocytic choriomeningitis virus infection of New Zealand black house mice, blockade of LTβ receptor reverses respiratory failure and immunopathology [19]. It is unknown if lymphotoxin is expressed in the lungs of deer mice infected with SNV. Conspicuous T lymphocyte migration into the lungs of infected deer mice is absent, suggesting that it is not, even though viral RNA can be detected more than 200 days post infection in some deer mice [8,9]. Antigen-specific T cells from the spleens of persistently-infected deer mice appear to be regulatory T cells [20], suggesting the lymphotoxin pathway is not used during persistence.

The TNF family is a frequent target of viral evasion strategies [21] and given the role of these proteins in inflammation they may also be targets of hantaviruses in their rodent hosts, which do not exhibit inflammatory responses. LT has been implicated in host-virus coexistence with human and mouse cytomegaloviruses by inducing IFNβ expression in fibroblasts without apparent cytotoxicity or induction of apoptosis [22]. In this manner, the expression of LT permits virus persistence without cytopathology. Since hantaviruses and rodent hosts have coevolved for tens of millions of years, it is likely that each has adapted to induce minimal pathology. Since deer mouse CD4^+ ^T cells are not susceptible to SNV infection [20] it is unlikely that viral proteins directly affect the LT pathways of these cells.

Structurally, deer mouse lymphotoxins are highly similar to orthologous polypeptides. Deer mouse LTα is predicted to be composed of 169 residues and LTβ is 302 residues long. Each polypeptide shares 11 secondary domains found in mouse and human lymphotoxins.

The deer mouse LTα and LTβ gene and polypeptide sequences are not conspicuously different from orthologous sequences. We did not obtain flanking information of the *Lta *gene, despite repeated attempts with primers designed to amplify additional 5' and 3' regions. These regions may be less conserved in deer mice compared to other rodent species, which would make primer design problematic. We were able to obtain some promoter information for the *Ltb *gene, which shares *cis*-elements found in the house mouse *Ltb *gene. One difference is the presence of an Sp1 *cis*-element in deer mouse. Whether this has functional consequences for *Ltb *expression will require further investigation since only lymphotoxin-α has been examined in human or rodent hantavirus infections [6,20]. All five of the promoters examined here also possess KLF *cis*-elements, which are recognized by a number of Sp1-family transcriptional repressors [23]. The intron/exon structure of deer mouse *Ltb *is identical to the other rodents, namely that all have three exons instead of four that is found in human *LTB *because of a splice donor site in intron 2 of the human gene. It is unusual, though, that while the deer mouse and house mouse have identical sequences at this site, the rat sequence possesses a three-nucleotide deletion. Deer mice (Sigmondontinae) are substantially more divergent than are rats and house mice (both Murinae) [24], suggesting the deletion in the rat gene occurred after the rat/house mouse divergence. Additional 5' and 3' information could provide information about these genes that is limited by our PCR approach. Alternative strategies for obtaining such information would be the use of genomic libraries, such as the recently-produced BAC library for the deer mouse . In addition, the deer mouse genome has recently been selected for sequencing (M. Dewey, pers. comm.), which should facilitate such analyses.

In agreement with many molecular studies on subfamily designations within Muridae [10,11,25,26], all of our analyses were able to resolve the separation between the Murinae and the Sigmodontinae. Future tests of LTα and LTβ utility need to add lymphotoxin sequence data from other muroid groups such as Tylonyinae, Neotomyinae, Cricetinae, and Arvicolinae; these subfamilies are particularly critical to sample because they are thought to be closely related to both the murines and the sigmodontines [11]. Although the analyses reconstructed Muridae, none of the data sets was able to ally the woodchuck (a member of the rodent family Sciuridae) with the other three rodents; in other words, the analyses were not able to resolve which one of the outgroups (woodchuck or human) was more closely related to the murid rodents. Further sampling within Sciuridae and other groups such as Gliridae and Dipodidae may be necessary to test the utility of these data for resolving family-level relationships within Rodentia. Despite that issue, the moderate level of useful variation that both LTα and LTβ possess, in combination with the high level of bootstrap support that characterized the subfamily-level lineages suggest that further investigation of phylogenetic utility is justifiable. In addition, because the two data sets may be combined with one another, investigators may have yet another approach at hand in resolving relationships that are currently problematic.

**Figure 6:**
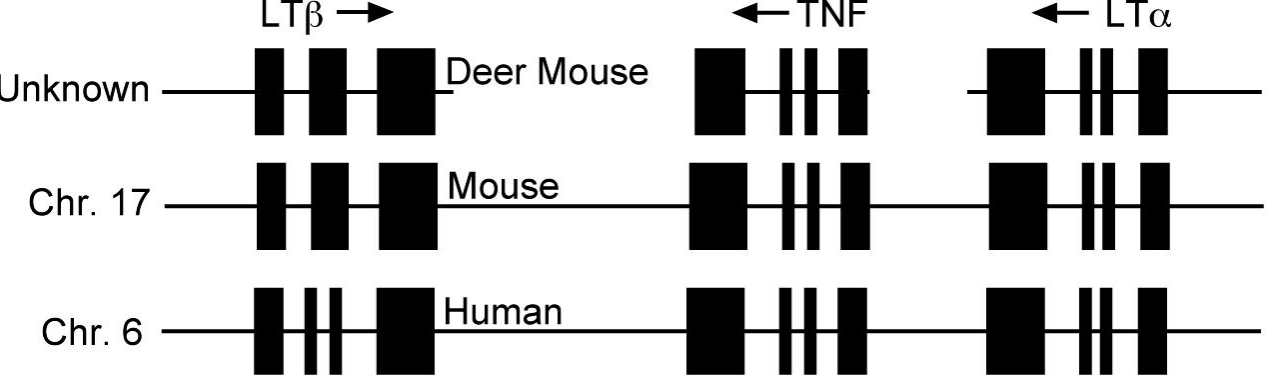
**Putative genomic organization of the deer mouse TNF locus.** The arrangement of the deer mouse genes is inferred from the TNF loci of the house mouse (chromosome 17) and human (chromosome 6) genomic regions. The blocks represent exons of each gene and the arrows indicate directionality.

Collectively, the tumor necrosis factor cluster is nearly identical between mammalian species (Fig. 6; [27]). While intervening sequences between *Tnf*, *Lta *and *Ltb *are not complete for deer mice, the structures of these genes are homologous between species. While unlikely, it is possible that gene orientation, order and location of the deer mouse genes are not syntenic to other species' TNF loci. The locus is found in the MHC in all mammalian species that have been examined; however, the chromosomal location of the deer mouse MHC has not yet been determined, but may reside on chromosome 21 (R. O'Neill, pers. comm.).

## Conclusion

These sequences represent the fourth rodent, and first New World murid, *Lta *and *Ltb *genes described thus far. They exhibit substantial sequence conservation to orthologous sequences and the availability of these sequences may provide insight into their functions in deer mice infected with SNV.

What potential do these molecules hold for phylogeny reconstruction and/or hypothesis testing? The results suggest that these molecules inform on the intrafamilial level within Rodentia; in addition, our findings indicate that combining the LTα and LTβ data into a single data set is appropriate. Thus, LTα and LTβ may hold promise for resolving subfamily relationships not only within Rodentia but within other mammalian orders as well. The implementation of broader taxonomic representation will allow for a fuller test of the utility of these molecules for phylogenetics.

## Methods

Liver DNA was extracted (PureGene DNA Isolation Kit, Gentra Systems, Minneapolis, MN, USA) from an adult deer mouse (*P. maniculatus sonoriensis*) [28] and used to amplify fragments representing the *Lta *and *Ltb *genes by PCR (PCR Core Kit, Qiagen, Valencia, CA, USA) with primers from highly conserved regions of mouse, rat and human orthologs. The fragments were cloned into pGEM-T Easy vector (Promega, Madison, WI, USA) and transformed into Novablue cells (Novagen, Madison, WI, USA). Plasmids containing inserts of the appropriate sizes were used for DNA sequencing. After initial sequencing runs using T7 and SP6 primers, insert-specific primers were used for primer-walking sequencing. The sequence files were assembled with Sequencher version 4 (GeneCodes, Ann Arbor, MI, USA) and submitted to blast to identify genes [29].

The *Lta *gene was amplified and cloned using a single primer set (forward, 5'-TGT CTT CCG CTG TGT GCC CC-3'; reverse, 5'-GTG AGA GCT CTG GGT CTG TTT GG-3'). The *Ltb *gene was cloned from 5' and 3' overlapping PCR products using primers designed from conserved orthologous sequences (5' end, forward 5'-TTC CCC AGT TCA GAG AAC C-3'; reverse 5'-TCC CTC TCC TGT AGT CCA CC-3': 3' end, forward 5'-GAG GCG AGC CAA GAA GAA GC-3'; reverse 5'-ACG ATT CAC ACA TTC GCA CC-3'). Promoter analysis was conducted using NIH Signal Scan database  (Prestridge, D.S. 1991).

### Phylogenetic Analyses

Phylogenetic analyses were conducted using the deer mouse nucleotide sequences collected in this study and all available orthologous sequences found in GenBank (for species names and accession numbers, please see table 1). For all of the analyses, sequences from the human, house mouse, rat, and woodchuck were accessed from GenBank. The sequences were aligned using default parameters in ClustalW [30]; the alignment was then checked and adjusted by eye. Regions that were difficult to align, because of their hypervariable natures, were removed from the final data sets. Before initiating the combined analysis, the two data sets were assessed for combinability using the incongruence length difference test [31]; the results of the test indicated that the lymphotoxin-α and lymphotoxin-β data sets were not significantly incongruent with respect to each other and could, therefore, be combined (P value = 1.000000). PAUP* (version 4.0b8) was used to perform maximum parsimony analyses for each set of nucleotide sequences [32]. All characters were weighted equally and because of the small size of each of the data sets, the exhaustive search option in PAUP* was used. Because of the low level of taxonomic representation found in the sequence databases for these molecules, outgroup designation was somewhat problematic. Nevertheless, the human and the woodchuck (a sciurid rodent) were chosen as outgroups for all of the analysis. Robustness of the recovered branches was evaluated using 1000 iterations of nonparametric bootstrapping [33].

**Table 1:** Accession numbers of sequences used in this work.

**Common name**	**Species**	**LTA**	**LTB**
Deer mouse	Peromyscus maniculatus	AY251294	AY282503
House mouse	*Mus musculus*	NM_010735	MMU16984
Rat	*Rattus norvegicus*	NM_080769	BX883046
Woodchuck	*Marmota monax*	AF095586	AF095587
Human	*Homo sapiens*	AY216498	AY216497

## Abbreviations

LT: lymphotoxin; LTα: lymphotoxin-α subunit; LTβ: lymphotoxin-β subunit.

## Authors' contributions

TR cloned and sequenced the *Ltb *gene; JP cloned and sequenced the *Lta *gene. AP conducted the phylogenetic analyses; TS provided the conceptual framework for the project and wrote the manuscript.
